# Gender and Racial/Ethnic Disparities in HIV Care and Viral Suppression at Jail Entry

**DOI:** 10.1007/s40615-024-02230-7

**Published:** 2024-11-27

**Authors:** Jocelyn T. Vaughn, Caryn E. Peterson, Jana L. Hirschtick, Lawrence J. Ouellet, Ronald C. Hershow, Sage J. Kim

**Affiliations:** 1 University of Illinois Chicago School of Public Health, Chicago, IL USA; 2 University of Michigan School of Public Health, Ann Arbor, MI USA

**Keywords:** HIV, HIV care engagement, Criminal justice, Health disparities, Intersectionality

## Abstract

Women and racial/ethnic minorities living with HIV are less likely than White men to be engaged in HIV treatment when entering US jails. Few studies have examined the intersection of gender and race/ethnicity among incarcerated populations. The **“**Enhancing Linkages to HIV Primary Care and Services in Jail Settings Initiative” (EnhanceLink) was a 10-site prospective cohort study of 1,270 people living with HIV in correctional facilities between 2008 and 2011. Using data from this study (N = 1,096), we assessed the likelihood of having a usual source of HIV care, utilizing ART, and viral suppression (HIV-1 RNA < 200 copies/ml) within 30 days of incarceration among the following groups, stratified by current gender and race/ethnicity, relative to non-Hispanic White men: Non-Hispanic Black women, non-Hispanic Black men, Hispanic/Latina (Hispanic) women, Hispanic men, and non-Hispanic White women. Compared to non-Hispanic White men, non-Hispanic Black women were 20% less likely to report that they had access to HIV care before incarceration after adjusting for age, sexual orientation, incarceration history, and medical comorbidities (prevalence ratio (PR) = 0.8, 95% CI: 0.7–0.9, *p* = 0.0002). Non-Hispanic Black, Hispanic, and Non-Hispanic White women were 30% less likely to utilize ART (respectively) than White men after adjusting for the same potential confounders (PR = 0.7, 95% CI: 0.6–0.9, *p* = 0.002; PR = 0.7, 95% CI: 0.5–0.9, *p* = 0.02; PR = 0.7, 95% CI: 0.5–1.0, *p* = 0.03). Our findings underscore the importance of culturally informed, community-based HIV interventions that promote equitable access to HIV care.

## Introduction

HIV and incarceration are highly convergent public health issues in the United States (US) [1]. The burden of HIV/AIDS in socially and economically disenfranchised populations, coupled with crime policies targeting these segments of society, have contributed to a prevalence of HIV in correctional settings that is approximately triple the national prevalence [2, 3]. In 2020, nearly 12,000 people with HIV (PWH) were in the custody of state or federal correctional authorities [4].

The dual burdens of incarceration and HIV are disproportionately borne by racial/ethnic minority groups, particularly non-Hispanic Blacks who represent approximately 42% of new HIV infections and one-third of persons in jails and prisons, despite comprising only 14% of the US population [4–6]. Non-Hispanic Black and Hispanic/Latina (hereafter referred to as Hispanic) women, while collectively representing only 29% of women in the US, comprise approximately 75% of HIV-infected women. Respectively, they are 1.7 and 1.3 times more likely to be imprisoned than non-Hispanic White women [6–8].

PWH who are incarcerated are less likely than PWH in the general population to be engaged in community-based HIV care and virally suppressed when they enter jail or prison [9]. Gender and racial/ethnic disparities in HIV treatment among incarcerated PWH also persist. Studies have shown that HIV-positive women and non-Hispanic Black jail detainees are significantly less likely to use ART and have worse clinical outcomes than their male, non-Hispanic White counterparts [10–13]. Intersectional identities are associated with even more profound disparities [14], and large studies of community-dwelling PWH have revealed disproportionately low levels of ART utilization and viral suppression and high HIV-related morbidity among Black and Hispanic women [15, 16].

Individual-level factors that have been found to influence HIV care utilization and viral suppression include substance use, mental health, housing, health coverage, education, and poverty [15–21]. Prior research suggests that barriers to care, which are endemic among PWH who interface with the criminal justice system [22, 23], may explain observed racial/ethnic and gender disparities in HIV and thus represent potential targets for public health intervention [15, 24]. Such barriers to care are distributed unevenly in PWH who are involved with the criminal justice system. Women enter jails with more social service needs, medical comorbidities, behavioral health concerns (e.g., substance use), and worse overall health than men, lending support to the argument that many women have distinct pathways to incarceration reflecting a lack of social and economic resources [13]. Some studies [15, 17, 18], although not all [19, 24], suggest that the primary drivers of HIV care outcomes may also vary by race/ethnicity and gender.

Knowledge about intersectional disparities in HIV is limited in part because most published studies of gender and racial/ethnic disparities have employed analytic approaches in which one social position is examined. Intersectionality theory, on the other hand, allows for the examination of health and disease at different intersections of identity and social positions, recognizing there may be important heterogeneity in effects and the mediating processes that reinforce inequities [25]. Intersectionality-informed methods have the potential to maximize relevance of findings to target communities through more precise measurement of these disparities and mediating pathways [25–27].

The** “**Enhancing Linkages to HIV Primary Care and Services in Jail Settings Initiative” (EnhanceLink) was a study of more than 1,200 PWH in US correctional facilities between 2008 and 2011. Our primary aim was to use data from this initiative to evaluate intersectional gender and racial/ethnic disparities in HIV care access and viral suppression among PWH entering jails. Our secondary aim was to assess mediating factors contributing to disparities in specific groups to inform interventions to reduce inequities. Based on the existing literature, we hypothesized that non-Hispanic Black and Hispanic women would be least likely to report having access to HIV care and to attain viral suppression at jail entry.

## Methods

### Study Population and Data

All data were collected as part of EnhanceLink, a 10-site prospective cohort study funded by the Health Resources and Services Administration (HRSA) as a Project of Special Significance. The details of the study have been described elsewhere [28]. Briefly, individuals who entered a partnering jail during the study period (2008–2011) opted in or out of HIV testing (depending on the jail) during the intake process. The primary aim of the project was to evaluate interventions that linked those who tested positive with community-based medical and support services after release. The jails were located primarily in large and moderately sized, centralized US cities [29] and clustered in the northeast, with a median HIV prevalence of 427 per 100,000 (IQR: 327–825 per 100,000) [30]. The data used for this analysis were collected upon or shortly after incarceration through in-person interviews except for viral loads and CD4^+^ T cell count, which were derived from jail-based medical chart review. The reporting period for all self-reported variables was the time of jail entry or the prior 30 days, except where otherwise noted.

A total of 1,270 incarcerated adults (≥ 18 years old) were enrolled in EnhanceLink between 2008 and 2011 and had complete baseline data. Because we were interested in patterns of treatment utilization among individuals with known HIV infection, we excluded those who self-reported being unaware of their HIV status before entering jail (n = 99). We also removed clients for whom current gender identity at incarceration (n = 5) or Hispanic ethnicity (n = 22) was missing, as well as non-Hispanic clients with no race information (n = 1). Individuals who reported that their race was not listed among the discrete options and self-identified as African (n = 10) or African American (n = 1) were classified as Black. Lastly, we excluded non-Hispanic participants with a racial identity other than Black or White (n = 14) and those who self-reported more than one race (n = 33) because these strata were too small for meaningful analysis. The resulting sample was 1,096, representing 86% of study clients with baseline information.

### Conceptual Model

**Fig. 1:**
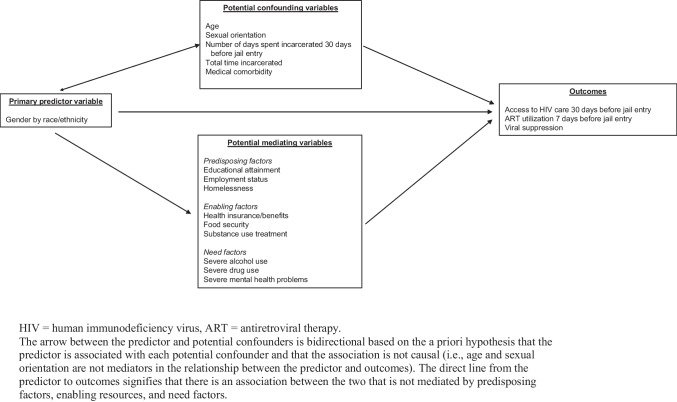
Conceptual model for the association between gender and race/ethnicity and HIV care outcomes, accounting for potential confounding and mediating variables

A conceptual model for the association of gender, race, and ethnicity with HIV-related outcomes is presented in Fig. 1.

To facilitate analysis, we organized potential mediating variables into three domains according to the Behavioral Model for Vulnerable Populations [31], a model that has been applied broadly in the literature and posits that healthcare utilization is determined by predisposing factors (e.g., homelessness), enabling resources (e.g., health insurance), and need (e.g., illness severity). Our model was informed specifically by prior studies in which investigators adapted the model for PWH [19–21, 32].

### Variable Definitions

#### Gender by Race/Ethnicity

Gender by race/ethnicity was cross classified based on self-reported gender, race, and ethnicity. It included six levels: non-Hispanic White (White) men, non-Hispanic Black (Black) men, Hispanic men, non-Hispanic White (White) women, non-Hispanic Black (Black) women, and Hispanic women, where those self-identifying as Hispanic could be classified as any race. Gender was measured dichotomously based on self-identification at incarceration; male-to-female transgender clients were classified as women (n = 20), and female-to-male transgender clients were categorized as men (n = 1).

#### Outcomes

We assessed two self-reported behavioral outcomes: Having access to HIV primary care during the 30 days before incarceration and any ART utilization within seven days before incarceration. Viral suppression was defined as HIV-1 RNA < 200 copies/ml. To capture clients’ viral status most effectively prior to the study-associated jail encounter, the value obtained most closely to and within 30 days of the incarceration (before or after) was analyzed. This approach rendered a sufficient sample size for stratified analysis with viral loads that theoretically approximate viral status at the time of incarceration [33].

#### Potential Confounding Variables

We selected potential confounders among the data elements available based on a priori hypotheses that each were: a) associated with the predictor, b) associated with the outcome, and c) not in the causal pathway. Dichotomous variables included self-described sexual orientation (classified as homosexual or bisexual vs. heterosexual), total time spent incarcerated over one’s lifetime, whether clients had been incarcerated in the 30 days before jail entry, and reporting at least one medical comorbidity among seven conditions that were evaluated during the baseline interview: Tuberculosis, hepatitis B, hepatitis C, asthma, diabetes, hypertension, and chronic pain. We included age as a continuous variable.

#### Potential Mediating Variables

We selected potential mediating variables among the data available based on the existing literature and conceptual models of HIV care utilization [15, 18–21, 32], as well as the degree to which characteristics could be modified (at least theoretically) through public health intervention. Predisposing factors included employment, which was classified as primarily unemployed or employed during the three years before incarceration. Our definition of homelessness aligned with previously published definitions [32]. We categorized educational attainment based on whether clients completed at least a high school degree or GED.

Enabling resources included having health insurance at the time of incarceration to pay for all or part of their medical care or medications. Food security was defined as having no more than a single day in the 30 days before jail entry in which they did not have food to eat [32]. Clients were classified as receiving substance use treatment if they received at least one day of outpatient treatment for drug or alcohol problems within the 30 days before incarceration.

Need factors included the presence of severe problems with drug use, alcohol use, and mental health. We measured these constructs by generating composite scores using the Addiction Severity Index (ASI) and previously published scoring methods [34, 35].

#### Other Variables of Interest

We assessed several additional variables not included in the conceptual model because we hypothesized that they were strongly associated with HIV-related outcomes. They included relationship status, receipt of public aid, the number of days spent incarcerated during the 30 days before jail entry, and mean CD4^+^ T cell count among those with values obtained ≤ 90 days before jail (n = 210). These variables were not included in regression models.

### Statistical Analysis

Differences in the distribution of study variables between gender-stratified racial/ethnic subgroups were tested using the chi-square test for categorical variables and the t-test or Wilcoxon rank sum test for normally and non-normally distributed continuous variables (respectively). The subgroup with the greatest prevalence or mean value for a particular variable served as the reference level for each comparison. We also assessed bivariate associations between all variables included in the conceptual model and the outcomes.

Modified Poisson regression [36] was conducted to assess differences in outcomes between each subgroup relative to the subgroup with the highest prevalence of the outcome. First, we measured crude differences in each outcome among subgroups. In the second model, we accounted for all potential confounding variables. We then assessed the contribution of potential mediating variables that might further account for differences in outcomes that remained statistically significant in a stepwise fashion, utilizing conceptual domains from the Behavioral Model for Vulnerable Populations. The variables included in each domain were determined separately for each predictor-outcome relationship by manual backward selection and a liberal alpha of *p* < 0.2 [37]. For example, all predisposing factors (education, employment, and homelessness) were added to the model collectively. The predictor with the largest non-significant *p*-value was dropped from the model, in decreasing order, based on an alpha level of *p* < 0.2. This procedure was repeated for enabling resources and need factors. After completing variable selection, we ran one fully adjusted model that included the variables meeting selection criteria from all three conceptual domains. We considered associations meeting an alpha level of *p* < 0.05 statistically significant. All analyses were performed using SAS, version 9.4 (SAS Institute Inc., Cary, NC) and R, version 4.2.3 (https://www.r-project.org/).

## Results

### Sample Characteristics

Of the 1,096 PWH in the sample, Black men were most highly represented (40.9%), followed by Hispanic men (20.5%) and Black women (18.2%) (Table 1). Clients had a mean age of 43.4 years (SD = 8.6), and approximately 22% self-identified as homosexual or bisexual. Most clients (71%) reported spending at least two years of their life incarcerated in jails and prisons, and nearly one in five (17.2%) spent some time incarcerated in a jail, prison, or hospital ≤ 30 days prior to the study-associated jail encounter.

**Table 1:**
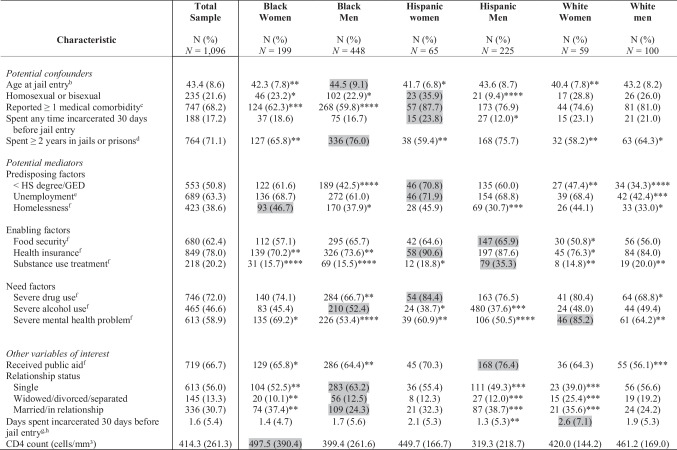
Selected Characteristics of PWH at Jail Entry, Overall and by Gender-Stratified Racial/Ethnic Subgroup—EnhanceLink, US, 2008–2011 (*N* = 1,096)^a^

PWH = people with HIV; HS = high school; GED = General Educational Diploma

^*^Statistically significant at *p* < 0.05; **Statistically significant at *p* < 0.01; ***Statistically significant at *p* < 0.001; ****Statistically significant at *p* < 0.0001

^a^The gray shading indicates the group with the highest prevalence or mean, which served as the reference group for the chi-square test or comparison of means

^b^Mean (SD), groups compared with the t-test

^c^Assessed based on whether they had any of the following medical comorbidities at jail entry: Tuberculosis, hepatitis B, hepatitis C, asthma, diabetes, hypertension, and chronic pain

^d^During clients’ lifetime

^e^During the three-year period before incarceration

^f^During the 30 days before incarceration

^g^Includes clients who did not spend any time incarcerated during the reporting period. Among the 188 clients who spent at least one day incarcerated, the mean number of days locked up ranged from 7.9 in Black women to 12.2 in Hispanic men

^h^Mean (SD), groups compared with the Wilcoxon rank sum test

Clients experienced a variety of social and structural barriers to care. Approximately 63% of clients reported that they were primarily unemployed during the past three years, half had not attained a high school degree/GED, and more than one-third (38.6%) were recently homeless. Approximately 72% and 47% of clients met criteria for severe drug use and alcohol problems, respectively, though only 20.2% reported receiving recent substance use treatment. Nearly sixty percent (58.9%) reported severe mental health problems.

Most clients had health insurance (78.0%) and were receiving public aid (66.7%) at the time of incarceration. However, there were relatively low levels of HIV care access. Among the overall sample, approximately 76.0% reported having a usual source of HIV care, 57.1% reported using ART, and 22.6% were virally suppressed.

**Fig. 2:**
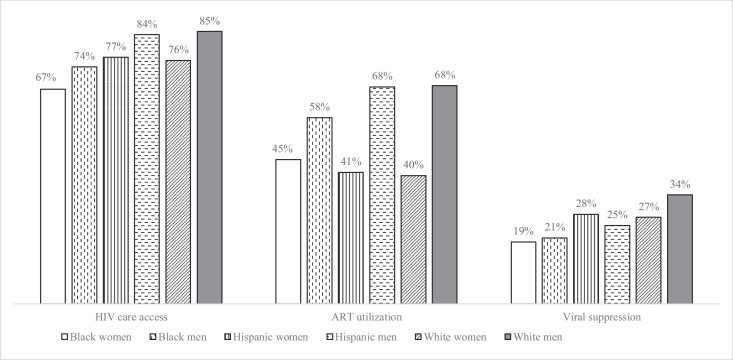
Comparison of study outcomes between gender-stratified racial/ethnic groups among PWH at jail entry—EnhanceLink, US, 2008–2011 (n = 1,096). HIV = human immunodeficiency virus; ART = antiretroviral therapy

There were meaningful intersectional differences in characteristics relevant to HIV care that would not have been evident had we stratified by gender or race/ethnicity alone. Hispanic women were the most likely subgroup to report a medical comorbidity (87.7%), lack a high school degree/GED (70.8%) and employment (71.9%), and report severe drug use (84.4%). Hispanic men also reported relatively high levels of severe drug use (76.5%) and greater access to substance use treatment than any other group (35.3%). Compared to other subgroups, Black women and men were less likely to report medical comorbidities (62.3% and 59.8%) and be insured (70.2% and 73.6%). Homelessness disproportionately affected women, ranging from 44.1–46.7% by race/ethnicity (vs. 30.7–37.9% in men). White women were significantly more likely than any other subgroup to report severe mental health problems (85.2%). White men were the most likely to have access to HIV care, utilize ART, and be virally suppressed (Fig. 2).

### Predictors of HIV Care Outcomes

Several individual-level factors were highly associated with HIV care and viral suppression in the overall sample (Table 2). Health insurance and food security were associated with greater ART utilization (prevalence ratio (PR) = 1.9, 1.5–2.4, *p* < 0.0001; PR = 1.4, 95% CI: 1.3–1.6, *p* < 0.0001), and insured clients were also twice as likely to be virally suppressed (PR = 2.0, 95% CI: 1.2–3.2, *p* = 0.008). Homelessness (PR = 0.7, 95% CI: 0.6–0.8, *p* < 0.0001) and severe drug use (PR = 0.8, 95% CI: 0.7–0.9, *p* < 0.0001) were negatively associated with ART utilization but not predictive of viral suppression. Clients with severe mental health problems and medical comorbidities, on the other hand, were 60% and 70% more likely (respectively) to be virally suppressed than their counterparts who did not report those challenges (PR = 1.6, 95% CI: 1.2–2.1, *p* = 0.004; PR = 1.7, 95% CI: 1.2–2.4, *p* = 0.006).

**Table 2:** Bivariate Associations Between Potential Confounders and Mediators with HIV Care Outcomes Among PWH at Jail Entry—EnhanceLink, US, 2008–2011

	Access to HIV care^a^ PR (95% CI)	ART utilization^b^ PR (95% CI)	Viral suppression PR (95% CI)
	*N* = 1,090	*N* = 929	*N* = 645
*Potential confounders*			
Age at jail entry^c^	44.2 vs. 41.3 (Ref)****	45.2 vs. 42.5 (Ref)****	45.6 vs. 43.6 (Ref)*
Homosexual or bisexual	1.1 (1.0–1.1)	1.0 (0.8–1.1)	0.8 (0.5–1.1)
Reported ≥ 1 medical comorbidity^d^	1.2 (1.1–1.3)***	1.1 (1.0–1.3)	1.7 (1.2–2.4)**
Days incarcerated 30 days before jail entry^a^	1.6 vs. 1.9 (Ref)	1.8 vs. 1.7 (Ref)	2.2 vs. 1.3 (Ref)
Spent ≥ 2 years in jails or prisons^c^	1.0 (0.9–1.1)	1.0 (0.8–1.1)*	0.8 (0.6–1.1)
*Potential mediators*			
Predisposing factors			
Homelessness^a^	0.8 (0.7–0.8)****	0.7 (0.6–0.8)****	0.9 (0.7–1.3)
Unemployment^e^	1.0 (0.9–1.0)	1.0 (0.9–1.1)	0.8 (0.6–1.1)
< HS degree/GED	1.0 (0.9–1.0)	0.9 (0.8–1.0)*	1.3 (1.0–1.8)
Enabling resources			
Food security^a^	1.1 (1.1–1.2)**	1.4 (1.3–1.6)****	1.0 (0.7–1.3)
Health insurance^a^	1.9 (1.6–2.2)****	1.9 (1.5–2.4)****	2.0 (1.2–3.2)**
Need factors			
Severe drug problem^a^	1.0 (0.9–1.1)	0.8 (0.7–0.9)****	1.1 (0.8–1.6)
Severe alcohol problem^a^	0.9 (0.9–1.0)*	0.9 (0.8–1.0)	1.0 (0.8–1.4)
Severe mental health problem^a^	1.0 (0.9–1.0)	0.9 (0.8–1.0)	1.6 (1.2–2.1)**
*Behavioral outcomes*			
Access to HIV care^a^	–	–	1.8 (1.2–2.6)**
ART utilization^b^	–	–	2.7 (1.9–3.9)****

PWH = people with HIV; HIV = human immunodeficiency virus; ART = antiretroviral therapy; PR = prevalence ratio; HS = high school; GED = General Educational Diploma

^*^Statistically significant at *p* < 0.05; **Statistically significant at *p* < 0.01; ***Statistically significant at *p* < 0.001; ****Statistically significant at *p* < 0.0001

^a^During the 30 days before incarceration

^b^During the seven days before incarceration

^c^Mean, groups compared with the t-test

^d^Assessed based on whether they had any of the following medical comorbidities at jail entry: Tuberculosis, hepatitis B, hepatitis C, asthma, diabetes, hypertension, and chronic pain

^e^During the three-year period before incarceration

### HIV Care Outcomes

#### HIV Care Access

Black women and men were 20% and 10% less likely (respectively) than White men to have access to HIV care (PR = 0.8, 95% CI: 0.7–0.9, *p* = 0.0002; PR = 0.9, 95% CI: 0.8–1.0, *p* = 0.01) (Table 3). After adjusting for potential confounders (age, sexual orientation, and incarceration history), differences remained significant in both groups (PR = 0.8, 95% CI: 0.7–0.9, *p* = 0.001 in Black women; PR = 0.9, 95% CI: 0.8–1.0, *p* = 0.04 in Black men). We found that adjusting for homelessness, health insurance, food security, substance use treatment, and severe mental health problems had no impact on the PR or 95% confidence interval for Black men, but the difference was no longer significant (PR = 0.9, 95% CI: 0.8–1.0, *p* = 0.09). After accounting for homelessness, health insurance, substance use treatment, and severe drug use, the disparity observed among Black women decreased slightly though remained statistically significant (PR = 0.9, 95% CI: 0.8–1.0, *p* = 0.04).

**Table 3:** Comparison of Access to HIV Care^a^ Between Gender-Stratified Racial/Ethnic Subgroups and White Men (Reference) Among PWH at Jail Entry—EnhanceLink, US, 2008-2011^b^

						Black women		Black men	
	N (%)	PR (95% CI)	*p*	PR (95% CI)^c^	*p*	PR (95% CI)^d^	*p*	PR (95% CI)^d^	*p*
	*N* = 1,090	*N* = 1,090		*n* = 1,020		*n* = 250		*n* = 480	
*Gender by race/ethnicity*									
Black women	131 (66.5)	0.8 (0.7–0.9)	0.0002	0.8 (0.7–0.9)	0.001	0.9 (0.8–1.0)	0.04	–	–
Black men	330 (74.0)	0.9 (0.8–1.0)	0.01	0.9 (0.8–1.0)	0.04	–	–	0.9 (0.8–1.0)	0.09
Hispanic women	50 (76.9)	0.9 (0.8–1.1)	0.21	0.9 (0.8–1.0)	0.17	–	–	–	–
Hispanic men	188 (83.9)	1.0 (0.9–1.1)	0.80	1.0 (0.9–1.1)	0.86	–	–	–	–
White women	44 (75.9)	0.9 (0.8–1.1)	0.18	0.9 (0.7–1.1)	0.22	–	–	–	–
White men	85 (85.0)	Ref	Ref	Ref	Ref	Ref	Ref	Ref	Ref
*Potential mediators* Predisposing factors									
Homelessness^a^						0.9 (0.8–1.1)	0.53	0.9 (0.8–1.0)	0.06
Unemployment^e^						–	–	–	–
< HS degree/GED						–	–	–	–
Enabling resources									
Food security^a^						–	–	1.0 (0.9–1.1)	0.79
Health insurance^a^						1.5 (1.2–2.0)	0.002	1.8 (1.5–2.2)	< 0.0001
Substance use treatment^a^						1.3 (1.2–1.5)	< 0.0001	1.1 (1.0–1.2)	0.03
Need factors									
Severe alcohol use^a^						–	–	–	–
Severe drug use^a^						0.9 (0.8–1.1)	0.07	–	–
Severe mental health problems^a^						**–**	**–**	1.0 (0.9–1.0)	0.34

HIV = human immunodeficiency virus; PWH = people with HIV; PR = prevalence ratio; HS = high school; GED = General Educational Diploma

^a^During the 30 days before incarceration

^b^White men served as the reference group for all regression models because they had the highest level of access to HIV care, which is theoretically attainable among other intersectional populations

^c^Model included potential confounding variables: age, sexual orientation, years spent incarceration during clients’ lifetime, medical comorbidities, and any incarceration ≤ 30 days before jail entry

^d^Model included potential confounding variables and potential mediating variables meeting inclusion criteria for the subgroups analyzed

^e^During the three-year period before incarceration

#### ART Utilization

Hispanic and White women were 40% less likely than White men to utilize ART (PR = 0.6, 95% CI: 0.4–0.9, *p* = 0.004; PR = 0.6, 95% CI: 0.4–0.9, *p* = 0.01), while there was an approximately 30% difference in Black women (PR = 0.7, 95% CI: 0.5–0.8, *p* = 0.0002) (Table 4). Adjusting for potential confounders did not substantially influence the estimates in any female racial/ethnic subgroup. After further accounting for homelessness, food security, health insurance, substance use treatment, and severe drug use, the difference in ART utilization in Black and White women narrowed slightly and remained statistically significant for Black women.

**Table 4:** Comparison of ART Utilization^a^ Between Gender-Stratified Racial/Ethnic Subgroups and White Men (Reference) Among PWH at Jail Entry—EnhanceLink, US, 2008-2011^b^

						Black women		Hispanic women		White women	
	N (%)	PR (95% CI)	*p*	PR (95% CI)^c^	*p*	PR (95% CI)^d^	*p*	PR (95% CI)^d^	*p*	PR (95% CI)^d^	*p*
	*N* = 929	*N* = 929		*n* = 876		*n* = 222		*n* = 129		*n* = 117	
*Gender by race/ethnicity*											
Black women	72 (45.0)	0.7 (0.5–0.8)	0.0002	0.7 (0.6–0.9)	0.002	0.8 (0.6–1.0)	0.03	–	–	–	–
Black men	215 (58.0)	0.9 (0.7–1.0)	0.05	0.9 (0.7–1.0)	0.09	–	–	–	–	–	–
Hispanic women	23 (41.1)	0.6 (0.4–0.9)	0.004	0.7 (0.5–0.9)	0.02	–	–	0.7 (0.5–1.0)	0.08	–	–
Hispanic men	140 (68.0)	1.0 (0.8–1.2)	0.98	1.0 (0.9–1.2)	0.80	–	–	–	–	–	–
White women	18 (40.0)	0.6 (0.4–0.9)	0.01	0.7 (0.5–1.0)	0.03	–	–	–	–	0.8 (0.5–1.1)	0.12
White men	62 (68.1)	Ref	Ref	Ref	Ref	Ref	Ref	Ref	Ref	Ref	Ref
*Potential mediators*											
Predisposing factors											
Homelessness^e^						0.8 (0.6–1.1)	0.12	0.8 (0.6–1.2)	0.37	0.8 (0.5–1.1)	0.21
Unemployment^f^						–	–	–	–	–	–
< HS degree/GED						–	–	–	–	–	–
Enabling factors											
Food security^e^						1.1 (0.8–1.4)	0.62	1.2 (0.9–1.7)	0.21	1.2 (0.9–1.6)	0.30
Health insurance^e^						1.7 (1.1–2.6)	0.01	1.5 (0.8–2.8)	0.17	–	–
Substance use treatment^e^						1.4 (1.1–1.8)	0.002	1.4 (1.1–1.9)	0.02	1.2 (0.9–1.5)	0.24
Need factors											
Severe alcohol use^e^						–	–	–	–	–	–
Severe drug use^e^						0.7 (0.6–0.9)	0.004	0.7 (0.5–0.9)	0.01	0.9 (0.7–1.1)	0.36
Severe mental health problems^e^						–	–	–	–	–	–

ART = antiretroviral therapy; PWH = people with HIV; PR = prevalence ratio; HS = high school; GED = General Educational Diploma

^a^During the seven days before incarceration

^b^White men served as the reference group for all regression models because they had the highest prevalence of ART utilization, which is theoretically attainable among other intersectional populations

^c^Model included potential confounding variables: age, sexual orientation, years spent incarceration during clients’ lifetime, medical comorbidities, and any incarceration ≤ 30 days before jail entry

^d^Model included potential confounding variables and potential mediating variables meeting inclusion criteria for the subgroups analyzed

^e^During the 30 days before incarceration

^f^During the three-year period before incarceration

#### Viral Suppression

Black women were 50% less likely than White men to be virally suppressed (PR = 0.5, 0.3–0.9, *p* = 0.03) (Table 5). Although the differences were marginally significant, we found that Black and Hispanic men were approximately 40% and 30% less likely (respectively) to enter jail virally suppressed (PR = 0.6, 0.4–1.0, *p* = 0.05; PR = 0.7, 0.4–1.1, *p* = 0.11). After adjusting for potential confounders, the disparity among Black women decreased by 40% and was rendered nonsignificant (PR = 0.7, 0.4–1.2, *p* = 0.19).

**Table 5:** Comparison of Viral Suppression Between Gender-Stratified Racial/Ethnic Subgroups and White Men (Reference) Among PWH at Jail Entry—EnhanceLink, US, 2008-2011^a^

	N (%)	PR (95% CI)	*p*	PR (95% CI)^b^	*p*
	*N* = 645	*N* = 645		*n* = 613	
*Gender by race/ethnicity*					
Black women	24 (18.6)	0.5 (0.3–0.9)	0.03	0.7 (0.4–1.2)	0.19
Black men	51 (20.5)	0.6 (0.4–1.0)	0.05	0.7 (0.5–1.2)	0.19
Hispanic women	12 (27.9)	0.8 (0.4–1.5)	0.49	0.8 (0.4–1.5)	0.50
Hispanic men	35 (24.5)	0.7 (0.4–1.1)	0.11	0.8 (0.5–1.3)	0.28
White women	8 (26.7)	0.8 (0.4–1.6)	0.50	0.9 (0.4–1.8)	0.75
White men	16 (34.0)	Ref	Ref	Ref	Ref

PWH = people with HIV; PR = prevalence ratio

^a^White men served as the reference group for all regression models because they had the highest prevalence of viral suppression, which is theoretically attainable among other intersectional populations

^b^Model included potential confounding variables: age, sexual orientation, years spent incarceration during clients’ lifetime, medical comorbidities, and any incarceration ≤ 30 days before jail entry

## Discussion

We found that among PWH entering jail, there were significant disparities in self-reported access to HIV care among Black persons and in ART utilization among women, relative to White men, that were not explained by differences in age, sexual orientation, prior incarceration, and medical comorbidities. Our findings illustrate the value of intersectional approaches to health disparities research versus independently examining one social category, such as gender or race/ethnicity. For example, the overall prevalence of reporting access to HIV care was 70% and 78% in women and men, respectively. However, there was important variation within gender groups, and disproportionately low access in Black women (67%). Similarly, while the prevalence of ART utilization was 54%, 62%, and 59% in Black, Hispanic, and White clients, there were stark gender differences among Hispanic men and women (68% vs. 41%, respectively). The overall pattern of unadjusted results across gender-stratified racial/ethnic groups that we observed are generally consistent with prior intersectional analyses of PWH, including the finding that Hispanic men were less likely to be virally suppressed than White men despite nearly equivalent levels of access to care [15, 16]. Although the reason for this gap is unclear, it is possible that Hispanic men initiated therapy later after diagnosis than White men, which is supported by prior research [38] and national surveillance data indicating that Black and Hispanic PWH are generally diagnosed at later stages than White PWH [39]. Enhancements to epidemiologic surveillance are needed to represent these intersectional risks among incarcerated PWH more completely.

We also identified factors that contributed to intersectional inequities. Health insurance, homelessness, and substance use related variables collectively accounted for small reductions in disparities among Black and White women and were strongly associated with outcomes in the overall sample, suggesting that these components are important prerequisites to care engagement. These findings reinforce the need for health care reform, including the expansion of comprehensive behavioral health services, to reduce health inequities among intersectional populations disproportionately affected by HIV, which are expected to substantially increase in size by 2060 [14]. The Affordable Care Act reduced barriers to HIV care and prevention by greatly expanding insurance coverage, and national data demonstrate that disparities in HIV prevention narrowed substantially since its implementation in some segments of the population [40]. However, coverage enhancements among PWH have largely occurred through Medicaid expansion [41], which has not been adopted in much of the US South where there is disproportionately high HIV burden. Increases in HIV testing between 2011 and 2018 did not benefit Black and Hispanic women like other groups [40]. The 21st Century Cures Act (Cures Act), signed into legislation in 2016, aims to improve health outcomes for marginalized populations in the US, including persons recently involved with the criminal justice system, while promoting a public health approach to treating substance use disorders. As previously noted [42], the Cures Act has the potential to alleviate racial/ethnic and gender-based health inequities if ample funding is devoted to addressing longstanding public health issues arising from past drug epidemics, like the crack epidemic of the 1980s, which largely affected predominantly Black, urban communities most vulnerable to discriminatory drug policies.

Although we identified factors contributing to HIV care access, the variables that we assessed did not fully account for inequities, and disparities among Black women remained statistically significant even after full adjustment, suggesting other mediating pathways were important. Analytic intersectionality acknowledges that social power shapes the causal processes that render health inequities in intersectional groups, and perceptions of these social positions lead to gender discrimination and racism that can impact care engagement and health [26, 27]. The strong associations we observed in Black women after accounting for the potential mediators in our conceptual model may signify that Black women in this population lacked social power in interpersonal and/or structural contexts preventing consistent access to HIV care. This view is consistent with prior research of Black women with HIV documenting strong associations between incarceration, violence-related service needs, and trading sex for goods [43]. Relatedly, HIV-associated stigma, fear, and lack of information are common among PWH, especially women [44, 45], and may have played an important role in the differences we observed. Further evaluation of discrimination and internalized stigma as causal processes mediating intersectional inequities in HIV is needed. Also, recognizing that PWH encounter multiple social and structural barriers to care that are influenced by positions of power and privilege, our findings lend support for interventions that empower persons to exercise agency in their own care and pair medical with social services [45, 46]. Research suggests that eHealth interventions may be more accessible and less stigmatizing than traditional, clinic-based interventions for PWH involved in the criminal justice system [47]. Alternatives to incarceration for substance use and other nonviolent offenses, such as drug courts, are also critical because they are associated with substantially less HIV treatment disruption and serve as effective venues for health interventions [48].

Our analysis had several limitations. First, data used for this analysis were collected between 2008 and 2011. While the results may not wholly reflect HIV care engagement among incarcerated populations today, there is still evidence of substantial HIV-related inequities the US [14], and health and health care in many US jails remains poor [49]. There are also limitations in the accuracy of self-reported HIV care engagement [50], and one of our primary outcomes was reporting access to HIV care, an imperfect proxy for care utilization.

Another important limitation is the lack of pre-incarceration CD4^+^ T cell counts, which we excluded from regression models due to large amounts of missing data. At the time of the study, ART was recommended for individuals with an AIDS-defining illness or with a CD4^+^ T cell count ≤ 500 cells/mm^3^. The omission of this variable precludes our ability to capture the potential effect of US clinical guidelines or to assess whether demonstrated disparities were associated with more advanced CD4^+^ T cell count depletion. However, we found that CD4^+^ T cell counts did not differ significantly among subgroups with complete data, and the mean in each subgroup fell below 500 cells/mm^3^.

Although analyses were adjusted for age, sexual orientation, and incarceration history, these variables were not captured in the intersectional differences explored in this study, preventing us from drawing conclusions about the contributions of these social identities. Similarly, we did not evaluate the influence of transgender experience because there were too few transgender clients to analyze independently from cisgender clients. However, in our sample, transgender persons were more likely to report a usual source of HIV care, utilize ART, and attain viral suppression than their cisgender counterparts (data not presented), and prior research suggests that justice-involved transgender and cisgender PWH have relatively similar levels of HIV care engagement [11], leading us to conclude that our definition of gender likely did not contribute to a type-I error.

Despite these limitations, our analysis contributes new information to the field. To our knowledge, this is the first study to assess intersectional gender and racial/ethnic disparities in HIV care among PWH entering US jails. By employing an intersectional approach to gender and racial/ethnic disparities, we identified inequities in care utilization and viral suppression that would not have been evident otherwise. This study provides useful information to guide the provision of culturally informed HIV-related programs and services.
